# A Law of Nature

**Published:** 1882-03

**Authors:** Wm. Jefferson Guernsey

**Affiliations:** Philadelphia


### A LAW OF NATURE.

WM. JEFFERSON GUERNSEY, M. D., PHILA.

If additional proof be needed to confirm the truth of our belief, that the maxim "similia similibus curantur" is based upon a law of nature, we find yet another illustration of the point in an able article on Hystero-Epilepsy by Chas. K. Mills, M. D., in the October number of the *American Journal of Medical Sciences.* One section thereof reads as follows: "Brown-Séquard has shown that some lesions of the spinal cord, of the medulla oblongata and of the nerves, especially the sciatic, will determine, in lower animals, the production of an affection in which manifestations, which approach closely those of epilepsy, show themselves a certain number of days after experimental traumatism. These animals, thus rendered epileptic, are sometimes attacked with convulsions spontaneously; but it is possible also to provoke these attacks by exciting a certain region of the skin which Brown-Séquard designates as the epileptogenic zone. This zone, situated on the same side of the body as the nervous lesion, has its seat about the angle of the lower jaw, and extends toward the eye and the lateral region of the neck. The skin of this region is a little less sensitive than that of the opposite side, but touching it most lightly provokes epileptic convulsions. The simple act of breathing or blowing on it brings about the same result. Something analogous to this epileptogenic zone has been noticed among hystero-epileptics. Simply touching the region is sufficient to provoke an attack, and this is more easily done if near the time of spontaneous seizure. After the grave attacks, the excitability seems to be exhausted, and pressure in the zone indicated does not cause any convulsive phenomena. In some cases these zones are double—it is necessary to touch two symmetrical points in order to bring on the convulsion. Touching but one has no effect in these cases. These zones occupy diverse points of the skin and of the deeper-seated parts, and, if they vary in different patients, they always occupy the same place in the same case. They are found on the trunk exclusively, more frequently in front than behind; in front they occupy lateral positions and are double and symmetrical; behind they are more often single and median. They exist more frequently to the left than the right, and the unilateral zones have always been met with on the left side. Ovarian pressure gives rise to spasmodic attacks; *the same pressure arrests them.* What is true of ovarian compression is equally true of all hysterogenic zones. A slight touch brings on the convulsions, which have scarcely commenced when they *can be stopped by a new excitation of the same point.* When a patient possesses several hysterogenic zones, the attack occasioned by an exciting of one *can be arrested by acting upon another.*"

The treatment of frost-bite with snow, of burns by heat, and inflammation by hot fomentations, are daily practices in the allopathic school, and although crude in their way, are, nevertheless, more successful than any other means known to them, and certainly homœopathic in principle, as also is the temporary relief of hystero-epileptic convulsions in the manner above cited. Why will not allopaths accept the suggestions of Dame Nature, and investigate a system of medicine based thereon?

